# Secondary Metabolites and Biological Activity of Invasive Macroalgae of Southern Europe

**DOI:** 10.3390/md16080265

**Published:** 2018-08-02

**Authors:** Patrícia Máximo, Luísa M. Ferreira, Paula Branco, Pedro Lima, Ana Lourenço

**Affiliations:** 1 LAQV-REQUIMTE, Departamento de Química, Faculdade de Ciências e Tecnologia, Universidade NOVA de Lisboa, 2829-516 Caparica, Portugal; lpf@fct.unl.pt (L.M.F.); paula.branco@fct.unl.pt (P.B.); 2 Sea4Us—Biotecnologia de Recursos Marinhos, Ltd., 8650-378 Sagres, Portugal; pedro.lima@sea4us.pt; 3 Nova Medical School/Faculdade de Ciências Médicas, Universidade Nova de Lisboa, Campo Mártires da Pátria, 1169-056 Lisboa, Portugal

**Keywords:** invasive species, *Asparagopsis* sp., *Caulerpa* sp., *Sargassum* sp., chemistry and biological activity

## Abstract

In this review a brief description of the invasive phenomena associated with algae and its consequences on the ecosystem are presented. Three examples of invasive algae of Southern Europe, belonging to *Rodophyta*, *Chlorophyta*, and *Phaeophyta*, were selected, and a brief description of each genus is presented. A full description of their secondary metabolites and biological activity is given and a summary of the biological activity of extracts is also included. In *Asparagopsis* we encounter mainly halogenated compounds. From *Caulerpa*, several terpenoids and alkaloids were isolated, while in *Sargassum*, meroterpenoids prevail.

## 1. Introduction

Alien species are plants, animals, or microbes that have been introduced and spread into new host regions, establishing populations that can become invasive if they interfere with the host ecosystem. These invasive species become established in natural or seminatural ecosystems, increasing in abundance and distribution and threatening biological diversity. They compete with native species, and usually have high reproductive rates assisted either by the lack of predators in the new environment or by the tolerance of a different range of environmental conditions. As a consequence, they are difficult to contain, harm biodiversity, and change the new host ecosystem [1].

Alien macroalgae are particularly likely to become invasive: their high reproductive rates, their production of toxic metabolites, and/or their perennial status make them more competitive than the native species, increasing the probability that they will become invasive. Several of these species periodically become a major problem, clogging waterways, fouling nets, and changing nutrient regimes in areas around fisheries, desalination facilities, and aquaculture systems [1]. They impact on local economies, such as fishery [2] and tourism. 

The mechanism of invasion by macroalgae thus begins with transport (by means of fouling, ballast waters, or aquaculture), proceeds by establishment of the species (through biotic and abiotic factors), and ends with its spread and impact [3,4,5,6,7]. Management of this update problem requires adequate measures [8] and control procedures, such as mechanical means, biological control, and/or chemical remedies [9].

With global warming there is a general increase of the tendency of invasive episodes, this being a situation of concern especially for Southern Europe. The Mediterranean coast and Atlantic areas near Gibraltar are key points in the dynamics and spread of these phenomena. As an example, in 2016, several beaches in Gibraltar were interdicted by Dictyota invasions with direct impact on local tourism, and remediation and management costs. However, macroalgae have underlying potential. Their commercial use as a source of nutraceuticals, food additives, biofuel, antifouling agents, or pharmaceuticals could be a way to exploit these phenomena in a more profitable way [4,10].

Thus, knowledge of the chemistry of these macroalgae is by no means out of date, as recent papers on the activity of algal extracts well document. Knowledge of their secondary metabolites and this review are also a starting point to the understanding of the chemistry of these species. There is a need, however, to fully characterize these invasive species in their new environment in order to make the most of their existence, and perform a strict correlation between metabolite and activity. 

In this review we chose three genera of invasive species of the Mediterranean—*Asparagopsis*, *Caulerpa*, and *Sargassum*—as examples of the chemistry of red, green, and brown algae, respectively. Two of them—*Asparagopsis* and *Caulerpa*—are already signaled by the International Union for Conservation of Nature (IUCN) Centre for Mediterranean Cooperation [1]. 

The secondary metabolites of the chosen genus are presented and, when possible, the studied biological activities are given. Reference to their study as invasive specimens is also provided. A list of reports on the biological activity of extracts is also given. This review covers the literature up to 2017.

## 2. Structural Characterization and Biological Activity

In this paper a chemical and biological activity summary of three different genera of invasive species of Southern Europe is presented. The structural identification of the mentioned metabolites relies on the usual techniques such as NMR, IR, MS, and chemical transformations for the less recent publications. Although some of the studies include biological activities of the isolated metabolites, most of the papers only mention isolation and characterization. 

### 2.1. Asparagopsis

*Asparagopsis* is a red seaweed genus of the family *Bonnemaisoniaceae* that has a diplohaplontic life cycle and a heteromorphic tetrasporophyte known as the “*Falkenbergia”* stage [11] Currently, only two species of this genus are accepted, *A. armata* and *A. taxiformis*, the former being endemic to the southern hemisphere and the latter being widely distributed in the tropics and subtropics [12]. Recently, a study of the lineages of this genus by DNA sequence was published [13].

Both species of this genus are native to Western Australia. *A. armata* is nowadays distributed throughout Europe in both the Atlantic and the Mediterranean basin, where it is highly invasive. *A. taxiformis* is invasive around the Indo-Pacific region, including Japan and Hawaii, and is currently widespread throughout the Mediterranean and along the Atlantic coast of Europe. While *A. armata* was probably introduced by maritime transport, *A. taxiformis* was probably introduced by oyster aquaculture [1].

*Asparagopsis* has been known to produce halogenated low-molecular-weight compounds [14,15,16,17,18,19,20,21].

**Figure 1:**
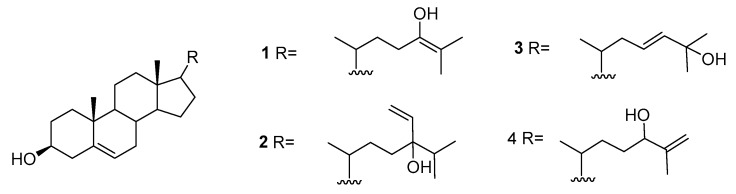
Hydroxylated sterols from *A. armata*.

We can also find reports on the presence of sterols in *A. armata* including 22-dehydrocholesterol, cholesterol, desmosterol, brassicasterol, 25-hydroxycholesterol, 25-hydroxy-24-methylcholesterol, fucosterol, β-sitosterol, liagosterol, and the hydroxylated sterols **1**–**4** represented in Figure 1 [22,23,24]. 

**Figure 2:**
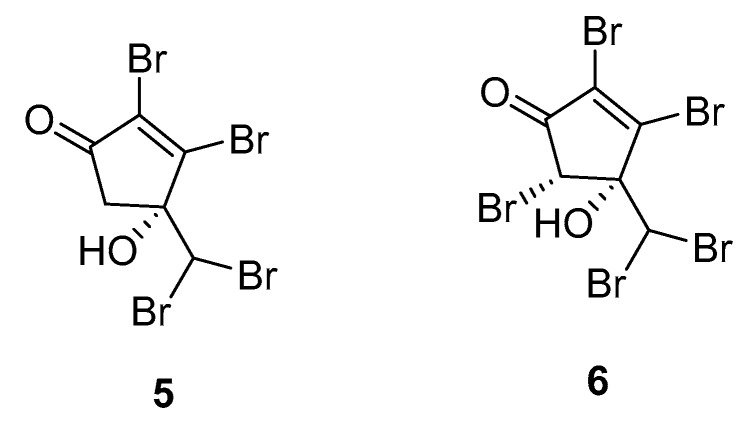
Brominated cyclopentenones from *A. taxiformis*.

A more recent study consists of the identification of the two brominated cyclopentenones **5** and **6** from *A. taxiformis* (Figure 2) [25]. 

Ecotoxicological activities of **5** and **6** against a marine bioluminescent bacterium (*Vibrio fischeri*) were used as an assessment of their role in the environment, revealing high toxicities for both compounds (EC_50_ effective concentration, 0.16 μM for **5** and **6**). Additionally, both compounds were evaluated in antibacterial, antifungal, and cytotoxicity assays. Compounds **5** and **6** exhibited mild antibacterial activities against the human pathogen *Acinetobacter baumannii*.

### 2.2. Caulerpa

Green algae of the genus Caulerpa Lamouroux represent the single genus in the family Caulerpaceae, which consists of approximately 60 species worldwide, generally distributed in shallow-water tropical and subtropical marine habitats. One of its species, *Caulerpa racemosa*, also known as “sea grapes”, is an edible marine green seaweed widely distributed throughout the South China Sea.

*C. racemosa* var. *cylindrica* is native to SouthWestern Australia, and is invasive in the Mediterranean [26,27,28] where its introduction is still speculative. Maritime traffic and aquarium trade are the most likely vectors. It can still be found in aquarium stores and is sold by internet retailers. *C. taxifolia* was accidentally introduced into the Mediterranean from a public aquarium in Monaco. Since then, it has spread rapidly due to its natural vegetative dispersal mechanism, its lack of natural grazers, and the ease of dispersion by boats, anchors, fishing nets, and aquaria [1].

We can find several reports on the chemistry of *Caulerpa* sp. These include the isolation of three squalene derivatives from *C. prolifera* [29] and fatty acids and sterols from *C. chemnitzia*, *C. faridii*, *C. manorensis*, *C. racemosa*, and *C. taxifolia*, including cholesterol, 24-methylcholesterol, 24-methyl-cholesta-7,22-diene-3β-ol, 4,24-dimethyl-cholesta-5,22-diene-3β-ol, and β-sitosterol [30]. 

**Figure 3:**
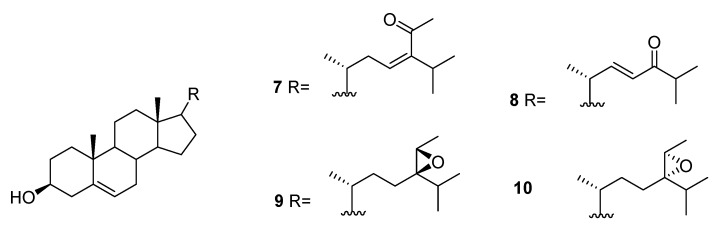
Oxygenated sterols from *C. racemosa*.

From *C. racemosa*, fucosterol and the oxygenated sterols **7**–**10** in Figure 3 were isolated, together with both C-24 epimers of saringosterol **2** [30,31]. 

**Figure 4:**
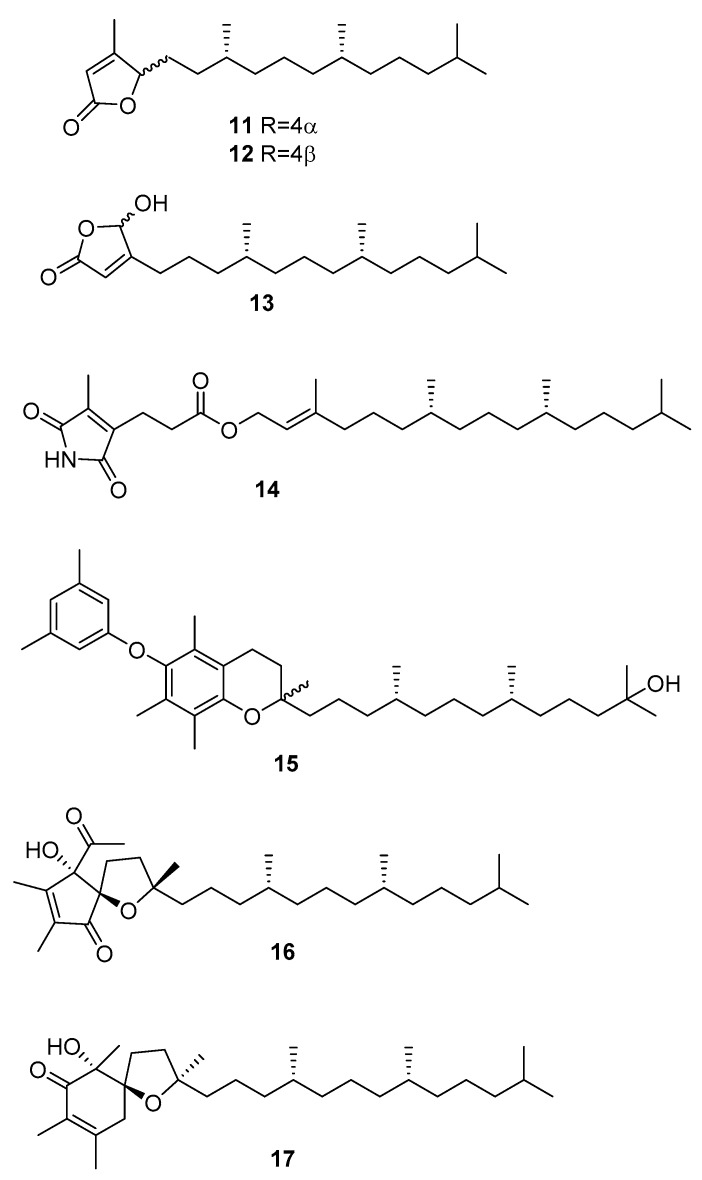
Metabolites from *C. racemosa.*

From *C. racemosa*, several varied metabolites were obtained by Yang et al. [31]. These include *trans*-phytol, *trans*-phytylacetate, α-tocopherolquinone, and the metabolites **11**–**17** in Figure 4.

The enzyme inhibitory activities of all the compounds were evaluated in vitro against PTP1B (protein tyrosine phosphatase 1B) and related PTPs (protein phosphatases) (TCPTP (T-cell PTP), CDC25B (cell division cycle 25 homolog B), LAR (leukocyte antigen-related phosphatase), SHP-1 (src homology phosphatase-1), and SHP-2 (src homology phosphatase-2)). Compounds **14**, *trans*-phytol, *trans*-phytylacetate, α-tocopherolquinone, **16**, and **17** and the sterols **7**, **8**, and 24*R* saringosterol **2** and **10** exhibited different levels of PTP1B inhibitory activity with IC_50_ (inhibitory concentration) values ranging from 2.30 to 50.02 μM. Of these compounds, **14**, α-tocopherolquinone, and **7** showed the most potent inhibitory activities towards PTP1B with IC_50_ values of 2.30, 3.85, and 3.80 μM, respectively. More importantly, the potent PTP1B inhibitors **14**, α-tocopherolquinone, and **7** also displayed high selectivity over the highly homologous TCPTP and other PTPs. The neuroprotective effects of the compounds against Aβ25–35 (amyloid β-peptide fragment 25–35)-induced cell damage in SH-SY5Y (neuroblastoma cell line) cells, a widely used neuroblastoma cell line for study of neurodegenerative disease, were also investigated. Compounds **17**, **7**, and **8** exhibited significant neuroprotective effects against Aβ25–35-induced SH-SY5Y cell damage with 11.31–15.98% increases in cell viability at 10 μM. In addition, the cytotoxic activities of the isolated compounds were tested against the human cancer cell lines A-549 (human lung carcinoma) and HL-60 (promyelocytic leukemia cells). Only the mixture of **11** and **12**, **16**, and α-tocopherolquinone exhibited moderate cytotoxicity against HL-60, and α-tocopherolquinone exhibited weak cytotoxicity against A-549 [31].

**Figure 5:**
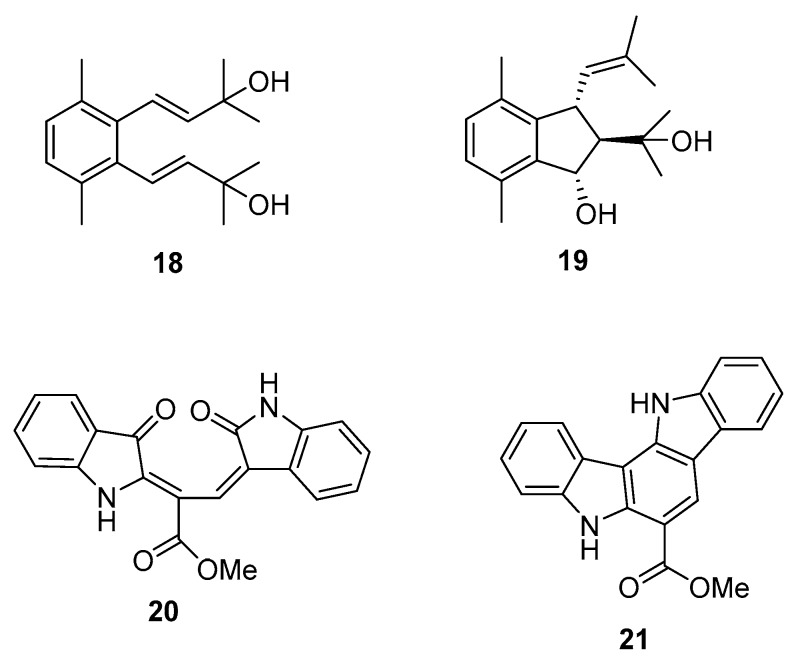
Metabolites from *C. racemosa*.

From *C. racemosa* we can also find two prenylated *p*-xylenes [32] **18** and **19** and racemosins A **20** and B **21** [33] (Figure 5). 

**Figure 6:**
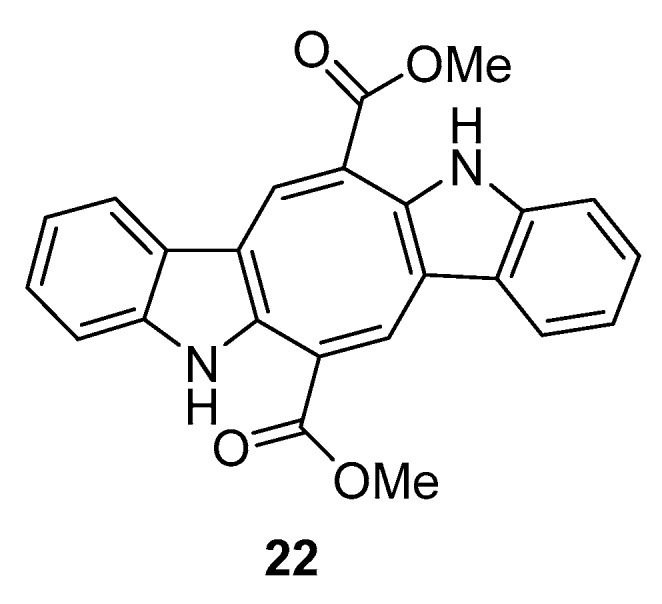
Metabolite from *C. prolifera*.

From *C. prolifera* [34], caulerpin **22** was isolated (Figure 6).

In in vitro bioassays, the compounds **18** and **19** exhibited a broad spectrum of antifungal activity against *Candida glabrata*, *Trichophyton rubrum*, and *Cryptococcus neoformans* with MIC_80_ (minimum inhibitory concentration) values between 4 and 64 μg/mL when compared to amphotericin B (MIC_80_ values of 2.0, 1.0, and 4.0 μg/mL, respectively) as a positive control and showed no growth inhibition activity against the tumor cells HL60 and A549 [32].

The biological activity of compounds **20**–**22** was tested in a neuroprotective bioassay using Aβ25–35-induced neurotoxicity in SH-SY5Y cells. Compound **22** showed significant neuroprotection (14.6% increase in cell viability) at the concentration of 10 μM, while compounds **20** and **21** showed moderate/weak neuroprotective activity with 5.5% and 8.1% increase in cell viability (10 μM), respectively, when compared to EGCG (epigallocatechin gallate), 16.57% increase at 10 μM) as the positive control [33].

**Figure 7:**
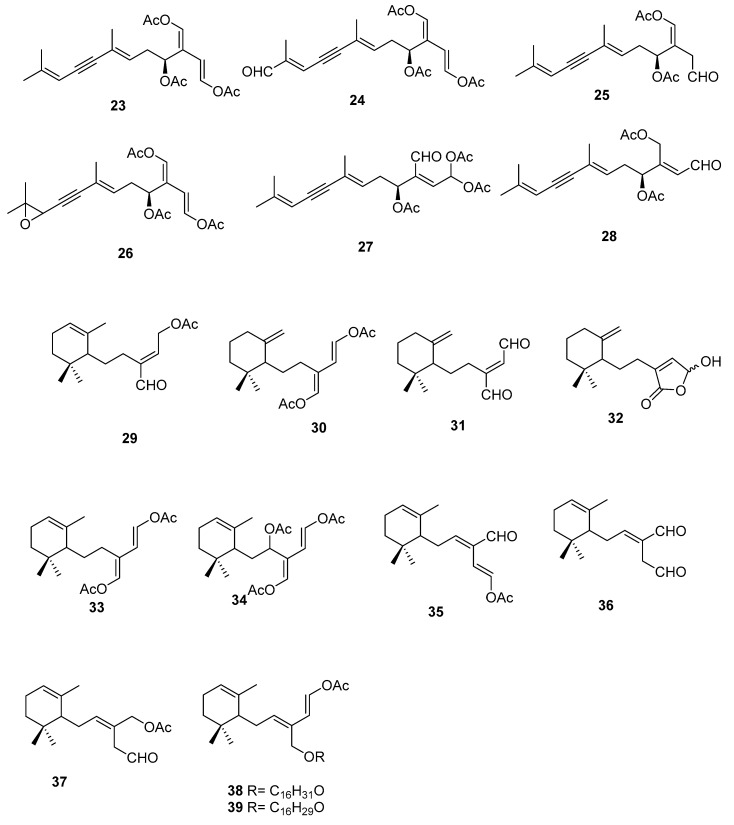
Sesquiterpenes from *Caulerpa* sp.

**Figure 8:**
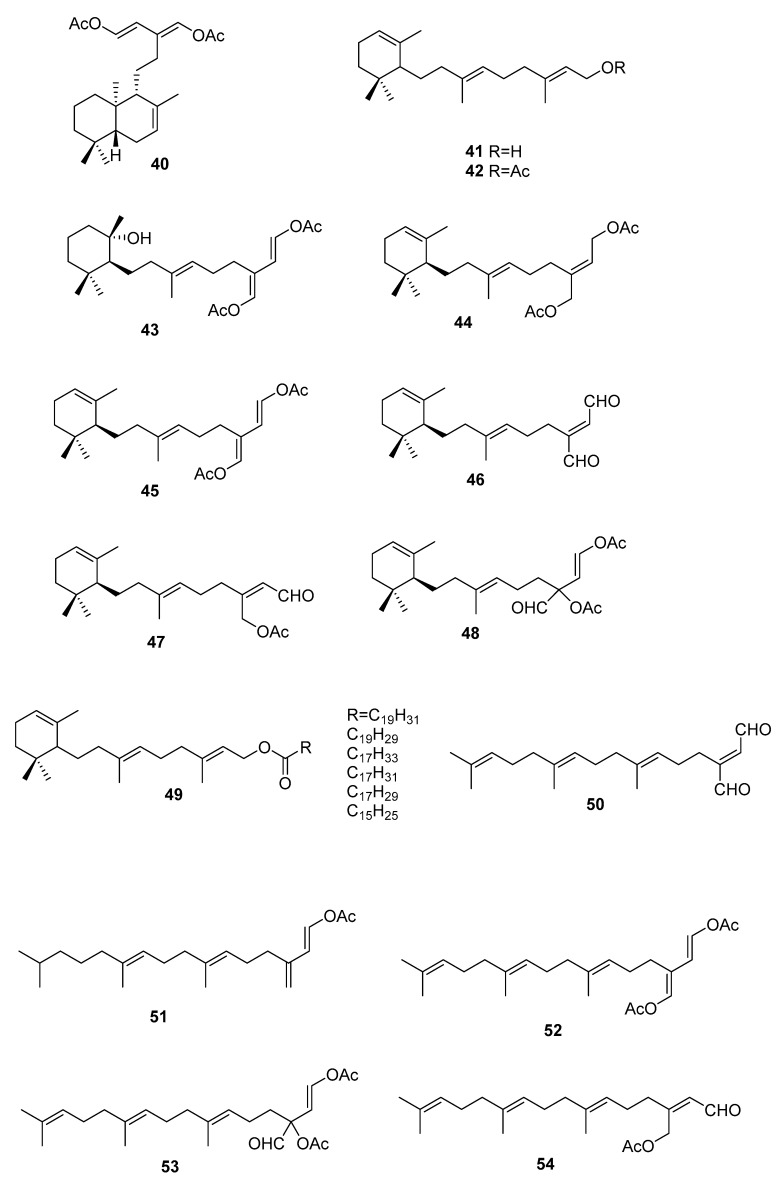
Diterpenes from *Caulerpa* sp.

On the terpenoid constituents of this genus we can find reports on monoterpenes [35,36]; the sesquiterpenes **23**–**39** (Figure 7, Table 1), isolated from several species [35,36,37,38,39]; the diterpene **40** from *C. trifaria* [40]; and the diterpenes **41**–**54** from *C. brownii* [41,42] (Figure 8).

A study [39] on the feeding preference by herbivorous fishes on several species of caulerpa led to isolation of **34**–**39** from *C. ashmeadii*. Compounds **34** and **36**–**39**, along with the alkaloid caulerpin **22**, were tested for field feeding preference, antimicrobial activity (against the marine fungus *Lagenidium callinectes*, and the bacteria *Vibrio* leignathi, V*. phosphoreum*, and SK13 (Gram-positive spore-forming bacteria requiring Mn for growth)), and ichthyotoxicity. All compounds except compounds **38** and **39** showed antimicrobial activity toward at least one marine bacterium. Compounds **36** and **37** also showed activity toward all three bacteria. All metabolites, except the fatty esters **38** and **39** and caulerpin **22**, were toxic to damselfish within 1.5 h. Compounds **36** and **37** again showed the highest degree of biological activity in this assay. 

From *C. bikinensis*, compounds **30**–**32** were isolated and tested as feeding deterrents [38]. The diacetate **30** and the dialdehyde **31** were found to be toxic to the Pacific damselfish *Pomacentrus phillipinus* at the 10 and 5 μg/mL levels. Feeding deterrence effects were reliably produced from **30** and **31** when tested at 1000 ppm levels against similar herbivorous fishes. The cytotoxicities of these compounds against the fertilized egg of the Pacific sea urchin *Lytechinus pinctus* were also measured. Again, **30** and **31** showed ED_50_ (effective dose) values of 2 and 1 μg/mL. The activities noted for these metabolites reinforce their likely roles in nature as agents of chemical defense. 

From *C. flexilis* var. *muelleri*, compounds **29** and **33** were isolated. No absolute configuration was determined for **33** [35].

From *C. prolifera*, **25** was isolated and its absolute configuration determined as *S* [37]. 

A study of *C. taxifolia* from Cap Martin, Côte d’Azur, at the time considered an invasive species, allowed the isolation of compounds **24**–**28**, for which no absolute configurations were determined. The proposed configurations were based on biosynthetic considerations [36].

From a larger study on algae of the order Caulerpales, diterpene **43** was isolated from *C. brownii.* [41]. Compound **43** had already been tested for biological activities. It showed antibacterial activity towards the pathogenic bacteria *Staphylococcus aureus* and *Bacilus subtilis*. It was also tested against marine bacteria and was found to be inhibitory towards *Vibrio harveyi* and *V. leiognathi*. It is also active against *E. coli* and *V. anguillarum* [41]. Handley reported the isolation of diterpenes **41**–**54** from branched and unbranched specimens of *C. brownii* and compound **50** was reported for the first time as a natural product [42].

From *C. trifaria*, diterpene **40** was isolated and the depicted configuration is proposed [40].

### 2.3. Sargassum

Sargassum is a genus of brown seaweeds with tropical and subtropical distribution, existing in all oceans. It is a large genus, comprising over 350 species. Some of its species are used in food in Japan and Korea, such as *S. fusiforme* and *S. muticum*. Due to air vesicles, *S. natans* and *S. fluitans* form large floating masses. *S. muticum* is invasive in the Mediterranean [43,44] and in Western Europe [45], and seems to have been introduced by the business of oyster culture [46].

A recent review on the therapeutic potential and health benefits of these species has been published [47].

**Figure 9:**
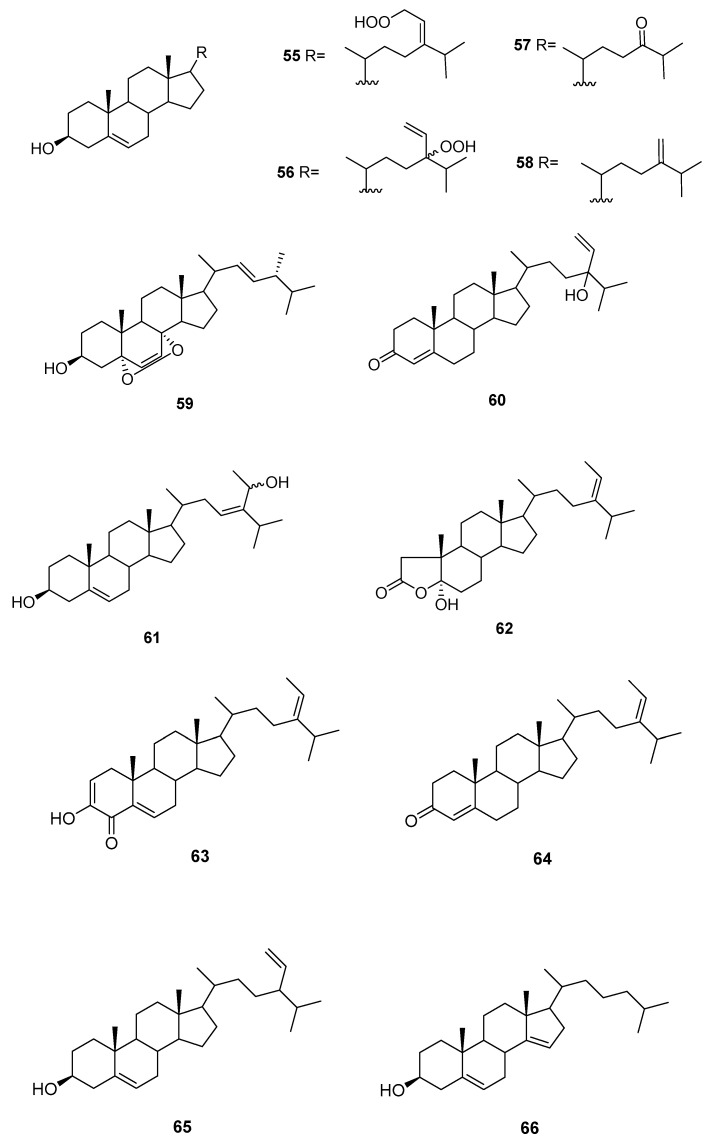
Sterols from *Sargassum* sp.

We can find several reports on the isolation of sterols (Figure 9) from *Sargassum* sp.

From *S. asperifolium* [48], saringosterol **2** and **60** were isolated.

From *S. carpophyllum* [49], **61** and **62** were isolated, together with fucosterol, 24-ethylcholesta-4,24(28)-dien-3,6-dione, **56**, **57**, **9**, and **10**. All compounds were tested for bioactivity of inducing morphological deformation of *P. oryzae* mycelia, and cytotoxic activity against several cultured cancer cell lines (P388 (mouse lymphocytic leukemia), HL-60, MCF-7 (breast adenocarcinoma), HCT-8 (human ilececal cancer), 1A9 (human ovarian cancer), HOS (human bone tumor), PC3 (human prostate cancer)).

The data showed that all the steroids exhibited activities causing morphological abnormality of *P. oryzae* mycelia. Fucosterol and 24-ethylcholesta-4,24(28)-dien-3,6-dione exhibited significant cytotoxicity toward P388 cancer cells, whereas **61** and **56** showed mild activity against the growth of HL-60 cancer cells. In the antitumor screen using a panel of human cell lines only the epoxy sterol **10** showed some cytotoxicity against several human cell lines. Compounds **62**, **9**, and **10** were also evaluated for HIV (Human immunodeficiency virus) growth inhibition activity in H9 lymphocytes. The EC_50_ and IC_50_ values for **9** were 0.500 and 0.975 mg/mL, whereas **62** and **10** were inactive.

From *S. fusiforme*, fucosterol [50,51], both C-24 epimers of saringosterol **2** [51] and **55**–**59** were isolated [51]. Fucosterol was shown to possess antidepressant and anticonvulsional effects [50]. Compounds **55**–**59**, fucosterol, and both C-24 epimers of saringosterol **2** were tested as LXR (liver X receptor) agonists: 24*S*-saringosterol **2** acted as a selective LXRβ agonist and was found to be potentially useful as a natural cholesterol lowering agent [51].

From *S. oligoscystum* [52], cholesterol, 22-dehydrocholesterol, fucosterol, both C-24 epimers of saringosterol **2**, and **55**, **56** and **58** were isolated.

From *S. thunbergii* [53], **63** was isolated, together with **3**, and **64**–**66**. Compound **63** exhibited significant inhibitory activity against human PTP1B with an IC_50_ value of 2.24 μg/mL.

From the genus Sargassum we can also find reports on the isolation of quinones and hydroquinones, chromenes, and varied structures.

Quinones and hydroquinones

**Figure 10:**
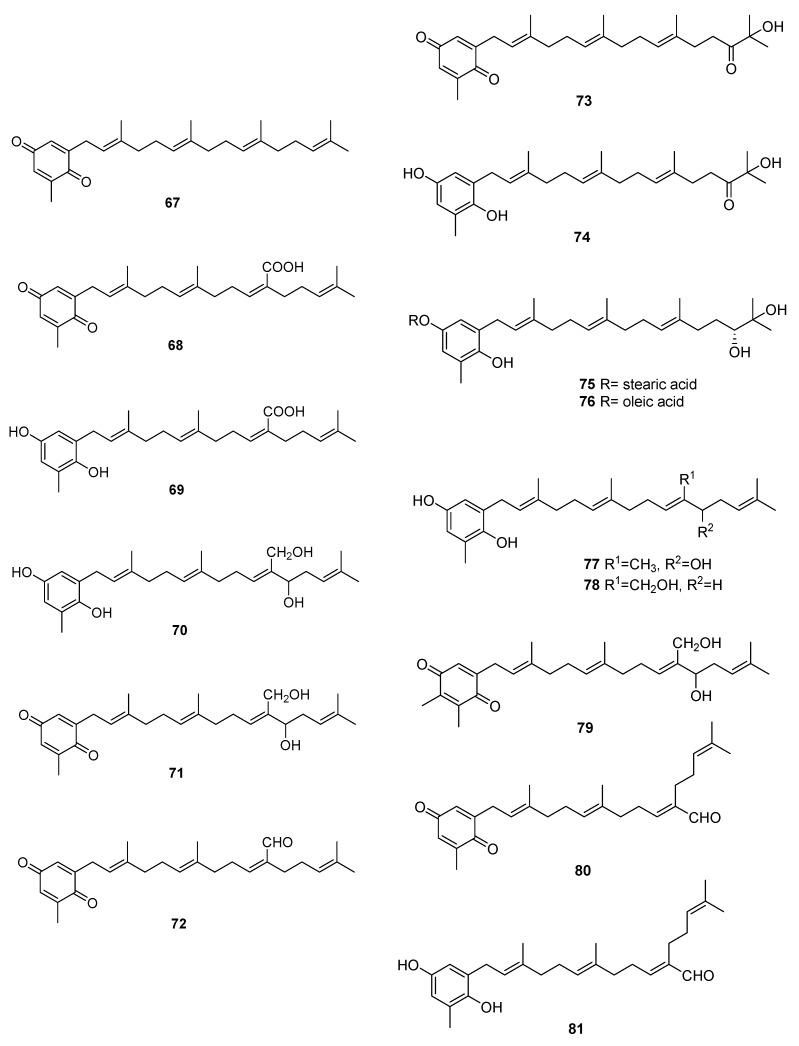

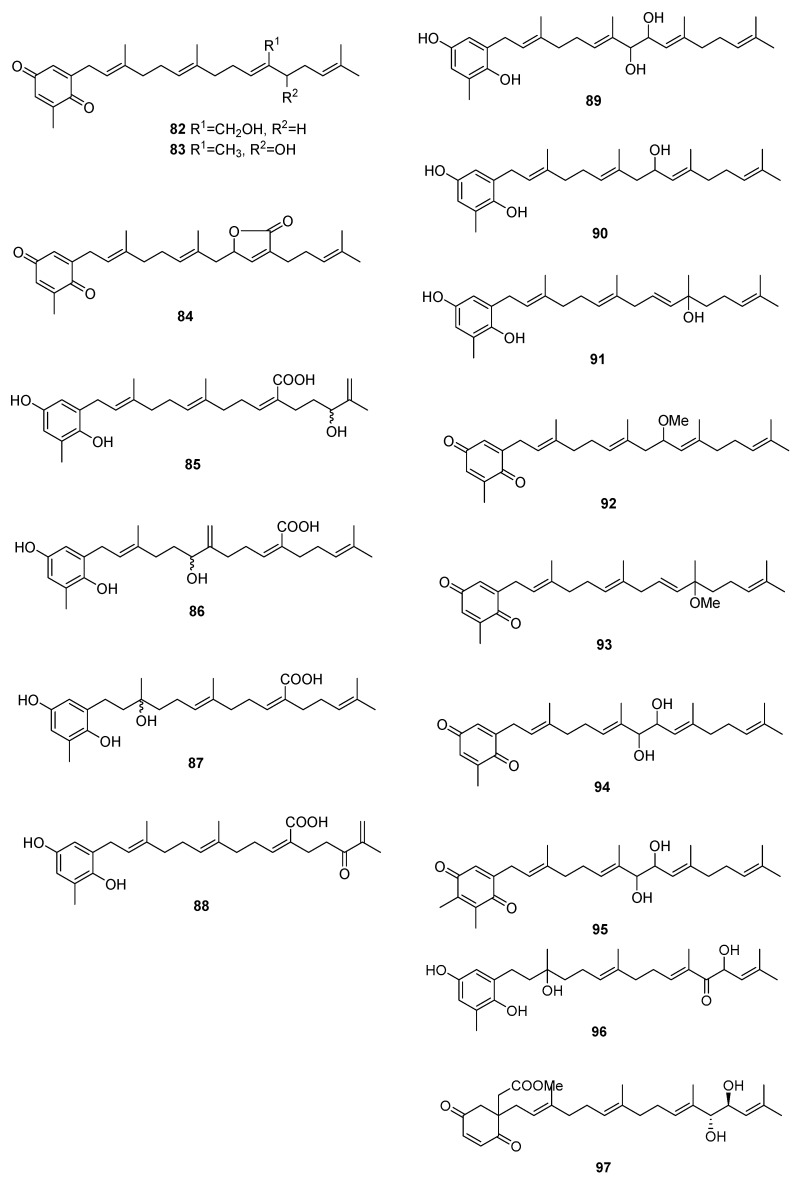
Quinones and hydroquinones from *Sargassum* sp.

We can find several reports on the isolation of quinones and hydroquinones from *Sargassum* sp. [54,55,56,57,58,59,60,61,62,63,64,65]. Their structures are in Figure 10 and occurrences are in Table 2.

From *S. elegans*, **68**, **69**, and **72** were isolated by electrochemistry-guided fractioning and their antioxidant potential was evaluated [54].

From *S. fallax* [55], **67**–**71** were isolated. Sargaquinone **67** was isolated as a mixture with sargaquinoic acid **68**. Both **68** and **69** were found to display moderate antitumor activity when tested against P388 cells. They displayed only weak activity against Bacillus subtilis.

From *S. herophyllum* [56], **67**, **69**, and **72** were isolated. They displayed moderate antiplasmodial activity against *P. falciparum*.

From *S. michranthum* [57], **73**–**76** were isolated. Compounds **74**–**76** displayed strong antioxidant activity, such as an inhibitory effect on NADPH-dependent lipid peroxidation in rat liver microsomes and radical-scavenging effect on DPPH (1,1-diphenyl-2-picrylhydrazyl). The inhibitory effect on lipid peroxidation was shown to be the same or stronger than that of the positive control, α-tocopherol. The authors identify the absence or presence of an unsaturated *cis* carbon–carbon double bond in the long-chain fatty acid ester moiety of **75** and **76** as responsible for the large difference in the inhibitory activity. Both compounds were found to have moderate radical-reducing effect on DPPH at a dose of each sample of 100 mg/mL. Based on these preliminary results, the author suggest that the hydroquinone moiety of **74** must participate in antioxidant activity, while in compounds **75** and **76**, hydrolysis of their ester group occurs first, and the resulting **74** may owe this activity. Antiproliferative activity of **74**–**76** against Colon 26-L5 cell was also evaluated. Compounds **74** and **76** showed relatively strong cytotoxic activity while moderate activity in the case of **75** was observed. 

From *S. paradoxum* [58], **67**–**71** together with **77**–**83** were identified by HPLC-NMR and HPLC-MS. Some of the compounds were isolated by bioguided fractioning and tested for their biological activity. Compared to the antibiotic ampicillin, the isolated compounds were far less potent against *S. aureus* and *S. pyogenes*. However, compounds **69**, **71**, **80**, and **260** were more potent against *P. aeruginosa* than ampicillin. There was no difference in activity between compounds with the hydroquinone or the *p*-benzoquinone moieties. The activity observed for sargaquinone **67**, the simplest of the meroditerpenoids isolated, suggests that the unsubstituted meroditerpenoid skeleton is responsible for the activity against *P. aeruginosa*. The addition of an alcohol group at position 12′ or 20′ (**70**, **77**, **78**, **82**, and **83**) appears to reduce the activity against *P. aeruginosa*, but increases the activity against *S. pyogenes*. Finally, incorporation of a carboxylic acid at position C-20′ (**69** and **68**) gives rise to activity against *S. aureus* and *S. aureus* MRSA Methicillin-resistant *Staphylococcus aureus*).

From *S. sagamium* var. *yezoense* [59], **68**, **69**, **80**, and **84** were isolated and from *S. sagamium*, **68** was isolated [64]. Its anticholinesterase activity and potential in Alzheimer’s disease is described [64]. The proapoptotic [65] and anti-inflammatory activities [69] of **68** are also documented. 

From *S. serratifolium* [60], **68** and **80** were isolated and from *S. siliquastrum* [61], **96** and **97** were isolated. Compound **96** showed radical-scavenging activity in DPPH assays.

From *S. thunbergii* [62,63], sargaquinoic acid **68** and sargahydroquinoic acid **69** were isolated. Since *S. thunbergii* was shown to inhibit adipogenesis in pre-adipocytes while enhancing osteoblast differentiation of pre-osteoblasts, and **68** and **69** were isolated in a bioguided study, the authors suggest that these two compounds possess osteoblastogenesis-enhancing abilities [63].

From *S. tortile* [66], **67** and **89**–**95** were isolated.

Compounds **68** and **69** were also isolated from *S. yezoense* [67]. Their effect on the transcriptional activity of PPARs (Peroxisome proliferator-activated receptors) was studied. The authors suggest that both compounds could be possible candidates for the treatment of type-2 diabetes and dyslipidemia. From *S. yezoense* [68], **85**–**88** were also isolated. Their antidiabetic potential was also evaluated.

### 2.4. Chromenes

**Figure 11:**
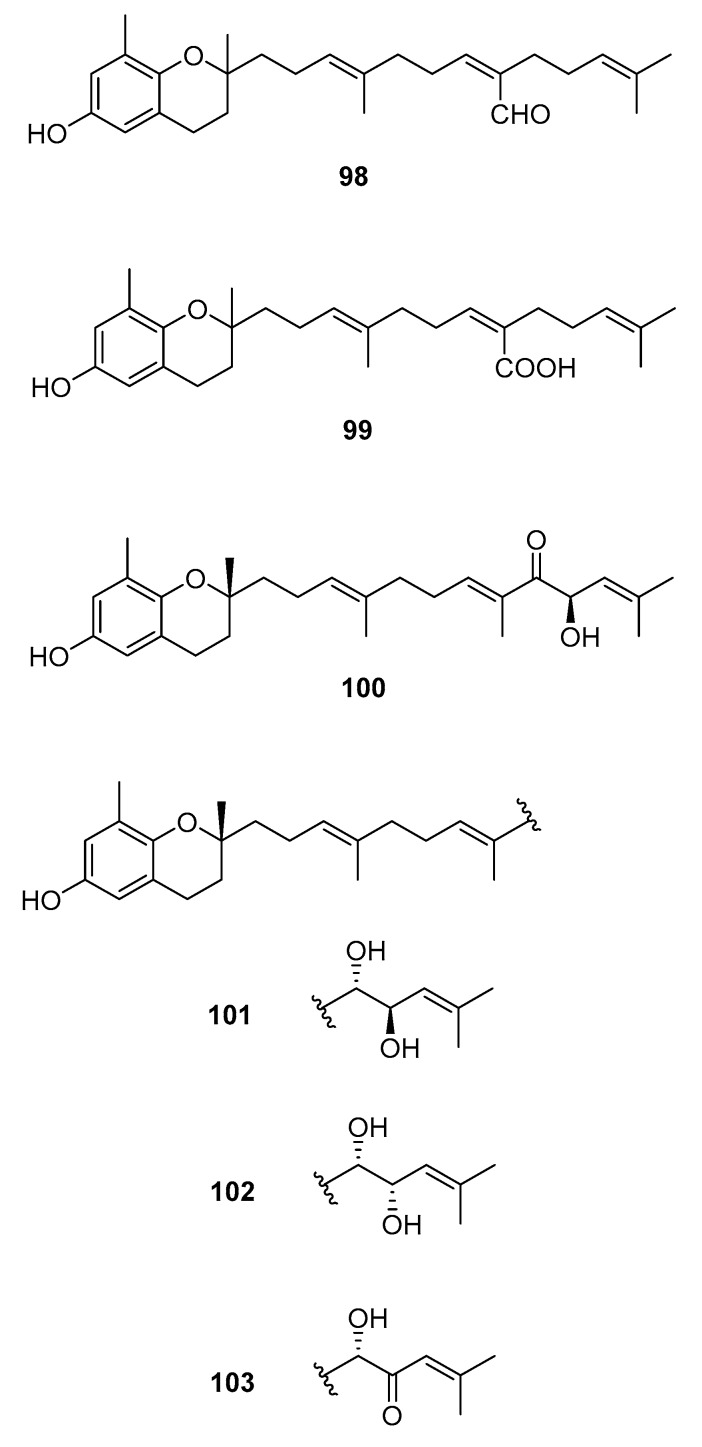

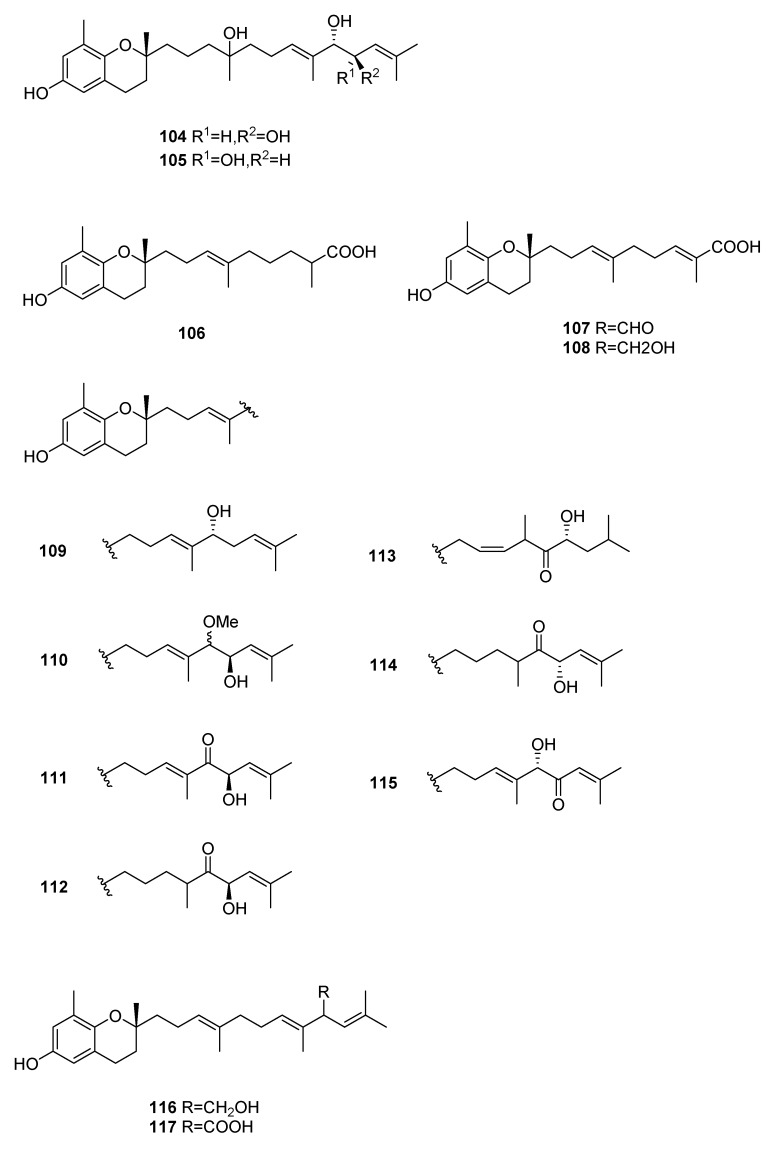

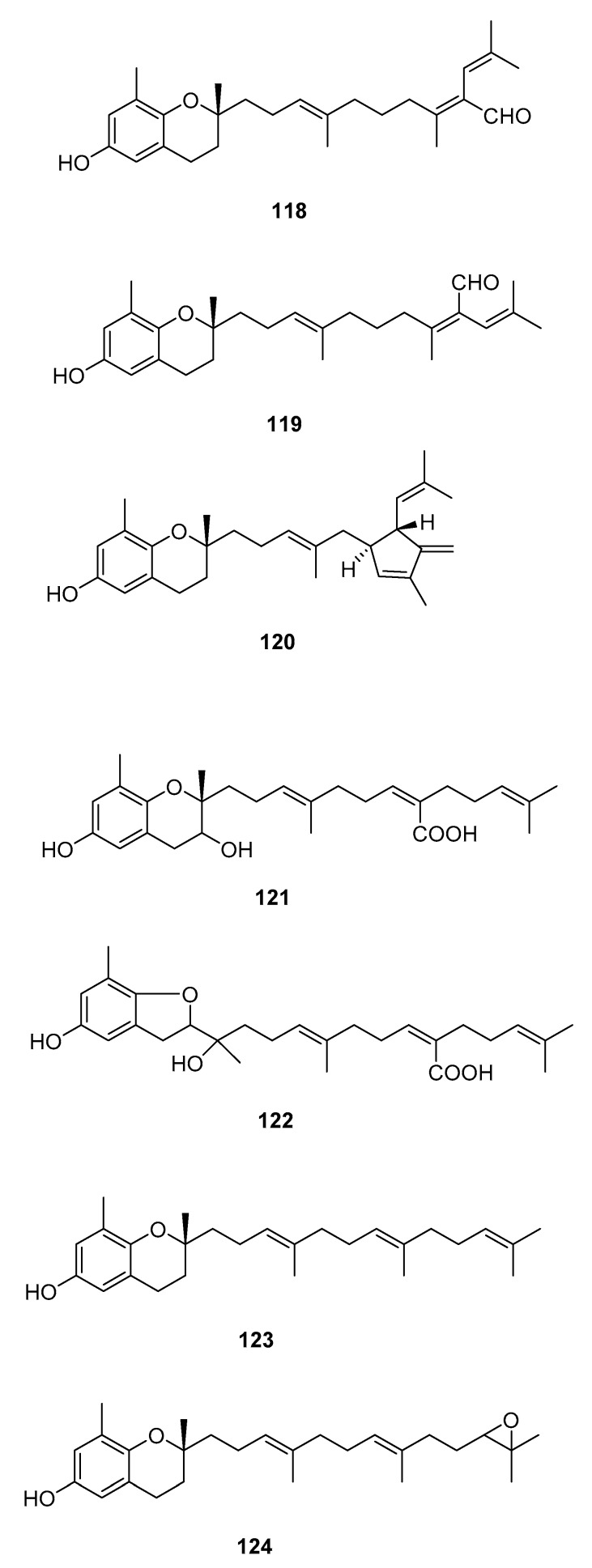

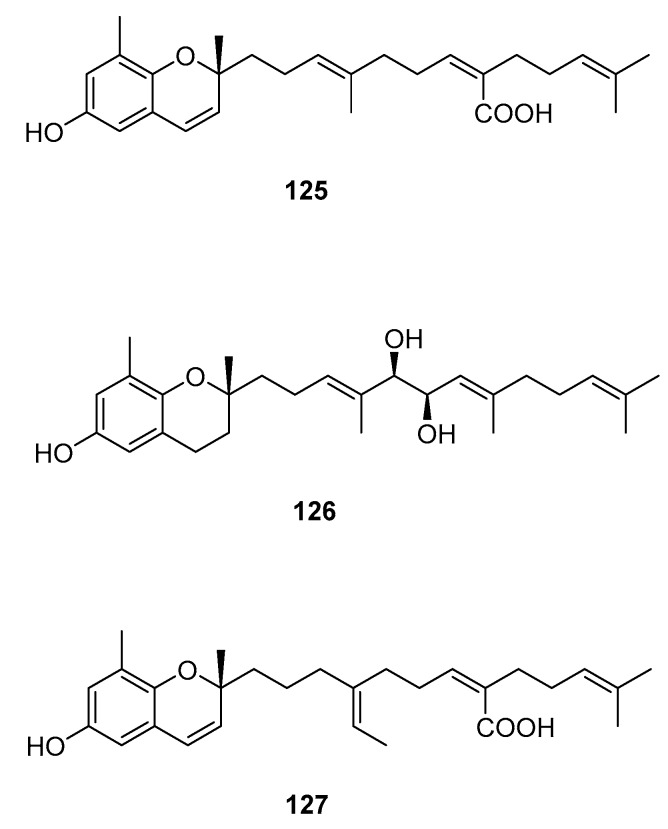
Chromenes from *Sargassum* sp.

We can also find reports on the isolation of chromenes [58,60,62,64,65,70,71,72,73,74,75,76,77]. Their structures are in Figure 11 and occurrences are in Table 3.

From *S. paradoxum* [58], **98** was isolated and from *S. serratifolium* [60], **99** was isolated. This compound was obtained from sargaquinoic acid **68** upon standing in methanol; it is therefore suggested to be an artifact.

From *S. sagamianum*, the isolation of **125** and its proapoptotic activity is described [65]. Its anticholinesterase activity and potential use in Alzheimer’s disease is also described [64]. 

From *S. siliquastrum*, Yoon [70] reported the isolation of **100**, and its potential as a novel anti-inflammatory agent was investigated. Lee [71] reported the isolation of **101**–**106**. The antioxidant activity of these compounds was evaluated by various antioxidant tests, such as scavenging effects on generation of intracellular ROS (reactive oxygen species), increments of GSH (glutathione) level, and inhibitory effects on lipid peroxidation in human fibrosarcoma HT 1080 cells. Compounds **101**–**106** significantly decreased generation of intracellular ROS and inhibited lipid peroxidation while they increased levels of intracellular GSH at a concentration of 5 μg/mL. Compound **101** was also isolated by Heo [72] and its anti-inflammatory activity against lipopolysaccharide-exposed RAW 264.7 cells was evaluated. Jang [73] reported the isolation of **101** and **102**, together with **107**–**120**. Although the configurations of **101**, **102**, and **120** are relative, for **109**–**115** the absolute configurations of the hydroxyl groups were determined by a Mosher’s method. Using DPPA (1,1-diphenyl-2-picrylhydrazyl), all of the compounds exhibited significant radical-scavenging activity in the range of 87–91% at the concentration of 100 μg/mL. In addition, compounds **111** and **117** displayed 82.7 and 80.0% inhibition, respectively, toward butylcholine esterase at the same concentration, while the other sargachromanols showed weaker or negligible activity. Cho reported the isolation of **127** and its antioxidant activity [77].

From *S. thunbergii* [62], **125**, **121**, and **122** were isolated. They were evaluated as to their capacity to scavenge DPPH radicals, and they exhibited EC_50_ values of 30 and 31 μg/mL, respectively, compared with BHT (butylated hydroxytoluene) (EC_50_, 32 μg/mL) and α-tocopherol (EC_50_, 18 μg/mL). On their scavenging activity on authentic ONOO^−^/induced ONOO^-^ from morpholinosydnonimine (SIN-1), their scavenging ratios on authentic ONOO^−^ were 60.0 and 57.1% at 5 μg/mL, respectively, while their inhibition ratios against the generation of ONOO^−^ from SIN-1 were 98.6 and 90.6% at the same concentration, respectively. Scavenging activities of L-ascorbic acid and penicillamine, positive controls, on authentic/induced ONOO^−^ were 98.1 and 90.4%, and 93.5 and 88.2%, respectively. 

From *S. tortile*, Kato [74] reported the isolation of **123** and **124**, together with their activity as attractants of the swimming larvae of *Coryne uchidai*. Kikuchi [75,76] reported the isolation and identification of **126**. Absolute configurations were determined by ECD (electronic circular dichroism). 

### 2.5. Other Compounds

**Figure 12:**
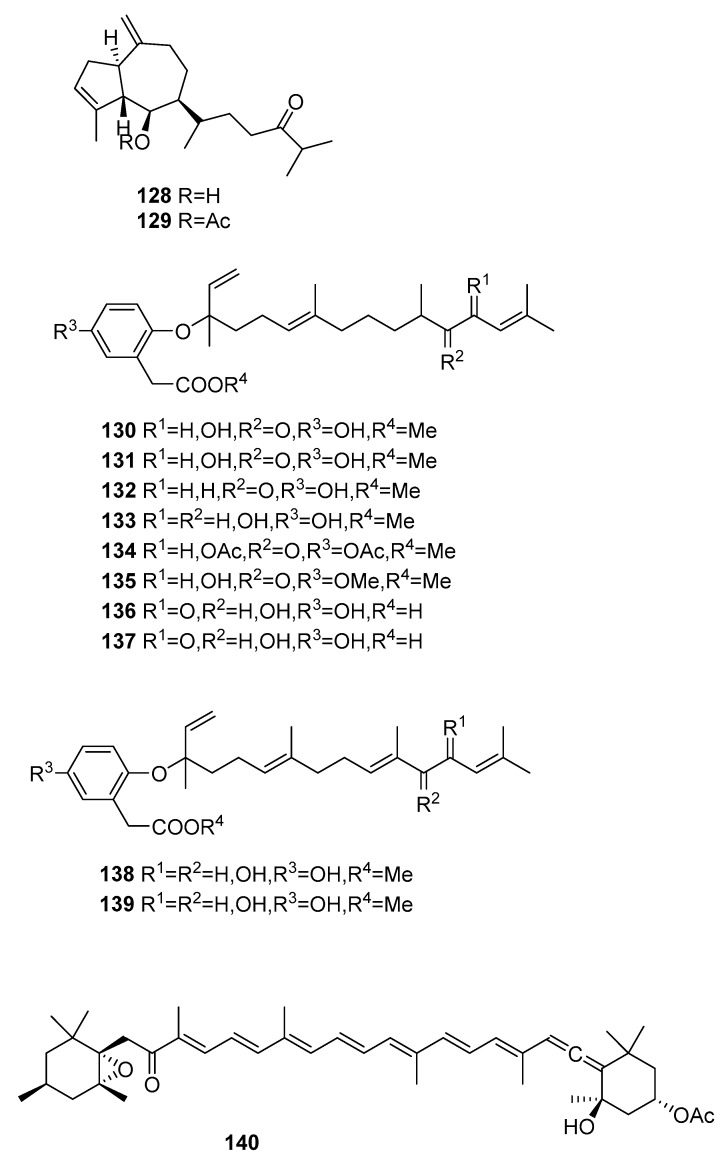

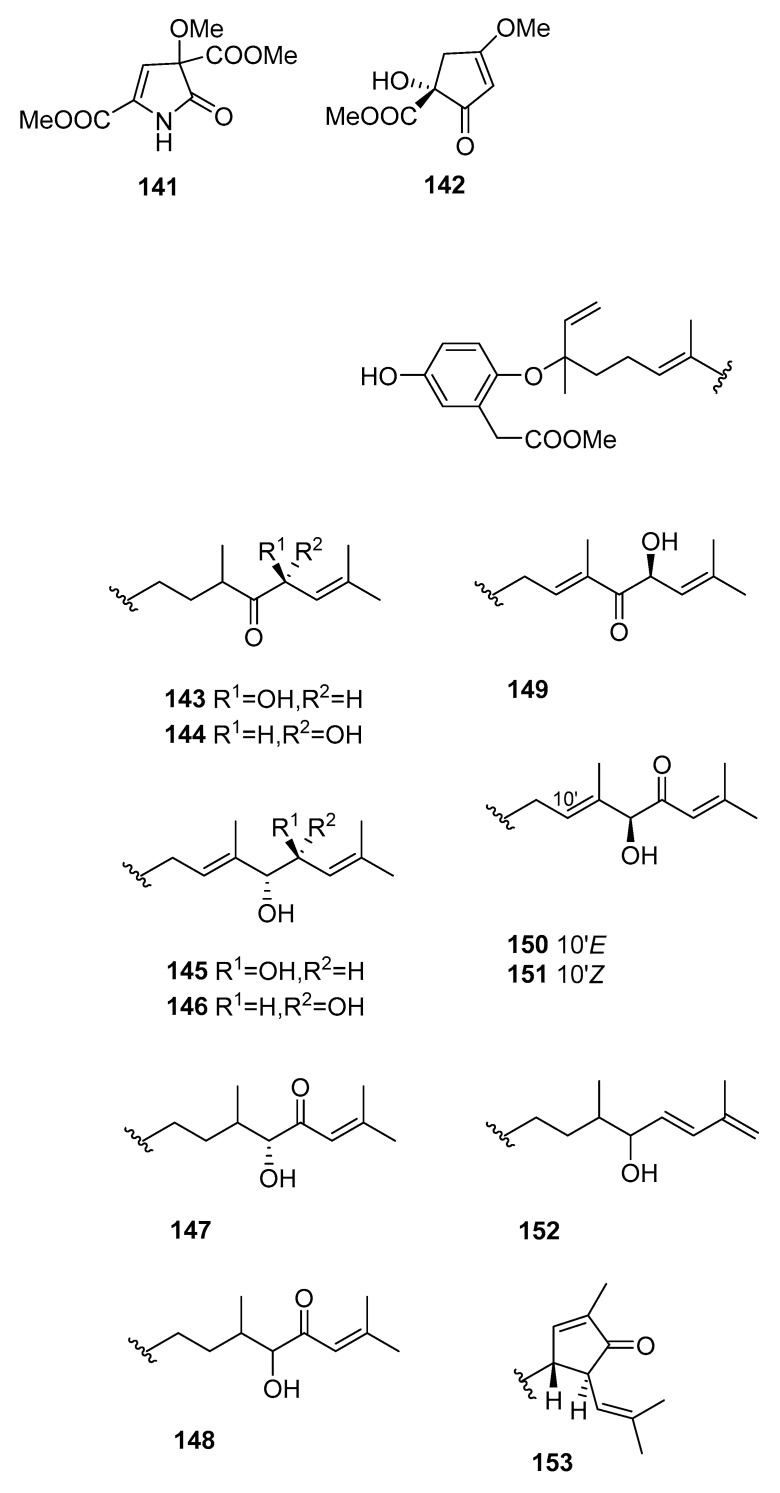

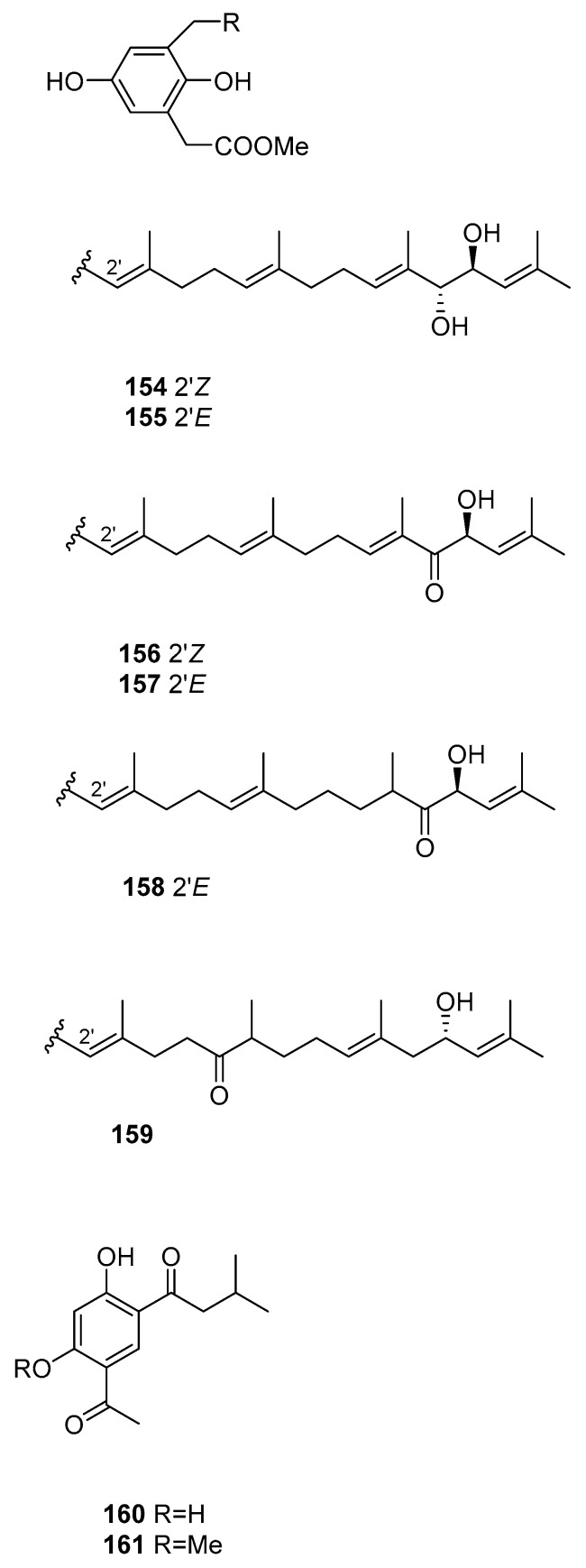
Other structures from *Sargassum* sp.

Within the constitution of *Sargassum* sp. we can also find various compounds [48,54,56,61,78,79,80,81,82]. Their structures are in Figure 12 and occurrences are in Table 4.

From *S. asperifolium* [48], two hydroazulenoids, **128** and **129**, were isolated.

From *S. autumnale* [78], compounds **130**–**139** were isolated and were tested as endothelin antagonists; they were not always potent and selective.

From *S. fusiformis* [79], fucoxanthine **140** was isolated by microwave-assisted extraction coupled with high-speed countercurrent chromatography. This compound was also isolated from *S. elegans* [54] and *S. heterophyllum* [56]. The antioxidant potential of **140** was evaluated [54] and it also showed a moderate antiplasmodial activity (IC_50_ = 1.5 μm) [56]. In order to assess the selectivity of fucoxanthin **140** for *P. falciparum*, the toxicity against a Chinese hamster ovarian cell line was evaluated. The relatively low cytotoxicity of fucoxanthin (IC_50_ = 83.7 μm) translated into a promising selectivity index (SI = antiplasmodial IC_50_/cytotoxicity IC_50_) of 54 [56]. From *S. Kjellmanium*, **141** [80] and **142** [81] were isolated. For both compounds, the structure was confirmed by single-crystal X-ray analysis.

From *S. siliquastrum* [61], compounds **143**–**159** were isolated. They showed moderate to significant radical-scavenging activity in DPPH assays. The 100-fold increase in radical-scavenging activity of the diphenolic isonahocols relative to the monophenolic nahocols indicated the role of the phenolic group in this activity. None of these compounds exhibited antimicrobial activity against Gram-positive or -negative bacteria or against pathogenic fungi. Conversely, the isonahocols **154**–**159** showed slight activity against sortase A derived from *Staphylococcus aureus*. The nahocols **143**–**153** showed no inhibitory activity against sortase A. These compounds were, however, weakly active against isocitrate lyase derived from *Candida albicans*.

From *S. thunbergii* [82], two resorcinols were isolated, **160** and **161**.

Finally, we can also find reports on the antifouling activity of fats and phthalic acid derivatives from *S. confusum* [83] and the isolation of farnesylacetones from *S.micracanthum* [84,85], from *S. sagamianum* with moderate anticholinesterase activity [86], and from *S. siliquastrum* with a moderate vasodilatation effect on the basilar arteries of rabbits [87]. Three linear bisnorditerpenes were also isolated from unidentified *Sargassum* sp. [88].

## 3. Biological Activity of Extracts

Macroalgae continue to attract the attention of researchers, as several reports on the activity of extracts in the literature testify. From the chosen genera here mentioned the following reports can be found.

### 3.1. Asparagopsis sp.

On the bioactivity of extracts from *Asparagopsis* sp. we can find reports on marine and biomedical antibacterial and antifungal activities of in both species of this genus [89,90,91,92,93,94,95,96,97]; nematicidal activity of *A. taxiformis* against the larvae of *Meloidogyne javanica* [98]; antifouling, anticyanobacterial, piscicidal, and crustacean toxicity of *A.taxiformis* [99]; and antioxidant and cytotoxic activities of *A. armata* [100].

### 3.2. Caulerpa sp.

For *Caulerpa* sp., studies on the bioactivity of extracts include antimicrobial activity of *C. occidentalis* [101], *C. cupressoides* [102], and *Caulerpa* sp. [103]; nematicidal activity of *C. racemosa* against the larvae of Meloidogyne javanica [98]; antioxidant activity of *C. lentilifera* and *C. racemosa* [104]; antinociceptive activity of *C. racemosa* [105], *C. mexicana*, and *C. sertularioides* [106]; anti-inflammatory activity of *C.mexicana* and *C. sertularioides* [106] and *C. peltata* [107]; antileishmania of *C. cupressoides* [102]; and antiviral activity against Dengue of *C. racemosa* [108] and HSV-1 (herpes simplex virus 1) of *C. cupressoides* [102]. Aqueous and methanolic extracts of *C. mexicana* were also found to suppress cell migration and ear edema induced by inflammatory agents [109].

### 3.3. Sargassum sp.

Reports on the bioactivity of extracts of *Sargassum* sp. include antifouling activity of *S. muticum* [110]; anticoagulant [111], antioxidant [112], and anti-inflammatory [113] activity of *S. horneri*; antioxidant activity of *S. siliquastrum* [114,115], *S. polycystum* [116], and *Sargassum* sp. [117]; antioxidant and anti-cholinesterase activity of *S. wightii* [118]; inhibitory effect on lipid peroxidation of *S. micracanthum* [119]; antimicrobial activity of *S. siliquastrum* [120]; antipyretic, analgesic, and anti-inflammatory *S. fulvellum* and *S. thunbergii* [121]; anti-inflammatory activity of *S. Serratifolium* [122]; antiallergenic activity of *S. tennerimum* [123]; anti-diabetic and hypolipidemic activity of *S. yezoense* [124]; larvicidal activity against malaria vector *Anopheles stephensi* of *S. swartzii* [125]; antigenotoxic activity of *S. dentifolium* [126]; antitumour activity of *S. wightii* against Dalton’s ascites lymphoma [127] and of *S. tenerrimum* against Ehrlich ascites carcinoma [128]; and antimelanogenesis activity of *S. polycystum* [129]. The action of *S. fulvellum* on skin dermatitis [130] and on neuronal maturation and synaptogenesis [131] is also documented, as well as the chemical genetic effects of *S. wightii* during embryonic development in zebrafish [132].

## 4. Conclusions

It is interesting to find the differences between the chemical compositions of all three genera. *Asparagopsis* is mainly rich in halogenated compounds, *Caulerpa* shows metabolites from varied biosynthetic routes, and *Sargassum* is rich in meroterpenoids. While biological activity of *Asparagopsis* metabolites is scarce, *Caulerpa* metabolites were shown to have inhibitory activity of PTPs, and to be neuroprotective, deterrents, and antibacterial. *Sargassum* metabolites are cytotoxic to cancer cells, and are antiplasmodial and antioxidants. Of course, only the more recent literature mentions biological activity results for the isolated metabolites. Extracts from all three genera show varied biological activities that make this a promising area of research. There is, however, a need to reinvestigate these genera as particular invasive species in their new host habitat since almost no reports are found on their chemistry. Their success in new environments can surely be correlated to their secondary metabolism and could provide new uses for otherwise noxious species.

## Figures and Tables

**Table 1:** Sesquiterpenes from *Caulerpa* sp.

Species	Compounds	Biological Activity
*C. ashmeadii* [39]	**34**–**39**	Feeding preference, antimicrobial, ichthyotoxicity
*C. bikinensis* [38]	**30**–**32**	Feeding deterrents
*C. flexilis* var. *muelleri* [35]	**29**, **33**	-
*C. prolifera* [37]	**25**	-
*C. taxifolia* [36]	**26**–**28**	-

**Table 2:** Quinones and hydroquinones from *Sargassum* sp.

Species	Compounds	Biological Activity
*S. elegans* [54]	**68**,**69**,**72**	Antioxidants
*S. fallax* [55]	**67**–**71**	Antitumour against P388
*S. herophyllum* [56]	**67**,**69**,**72**	Antiplasmodial activity
*S. michranthum* [57]	**73**–**76**	Antioxidants, radical scaveging, inhibitory effect on lipid peroxidation, antiproliferative against 26-L5, cytotoxicity
*S. paradoxum* [58]	**67**–**71**,**77**–**83**	Antibacterial
*S. sagamium* var. *yezoense* [59]	**68**,**69**,**80**,**84**	-
*S. sagamium* [64,65]	**68**	Anticholinesterase activity, proapoptotic, and anti-inflammatory
*S. serratifolium* [60]	**68**,**80**	-
*S. siliquastrum* [61]	**96**,**97**	Radical scavenging
*S. thunbergii* [62,63]	**68**,**69**	Osteoblastogenesis-enhancing abilities
*S. tortile* [66]	**67**,**89**–**95**	-
*S. yezoense* [67,68]	**68**,**69**,**85**–**88**	Transcriptional activity of PPARs (Peroxisome proliferator-activated receptors), antidiabetic potential

**Table 3:** Chromenes from *Sargassum* sp.

Species	Compounds	Biological Activity
*S. paradoxum* [58]	**98**	-
*S. serratifolium* [60]	**99**	-
*S. siliquastrum* Yoon [70,71,72,73,77]	**100**–**120**,**127**	Anti-inflammatory, antioxidant, radical-scavenging activity, inhibition of butylcholine esterase
*S. sagamianum* [64,65]	**125**	Proapoptotic activity, anticholinesterase activity
*S. thunbergii* [62]	**121**,**122**,**125**	Radical scavenging
*S. tortile* [74,75,76]	**123**,**124**,**126**	Larval attractants

**Table 4:** Other structures from *Sargassum* sp.

Species	Compounds	Biological Activity
*S. asperifolium* [48]	**128**,**129**	-
*S. autumnale* [78]	**130**–**139**	Endothelin antagonists
*S. elegans* [54]	**140**	Antioxidant
*S. fusiformis* [79]	**140**	-
*S. heterophyllum* [56]	**140**	Antiplasmodial, cytotoxicity
*S. Kjellmanium* [80,81]	**141**,**142**	-
*S. siliquastrum* [61]	**143**–**159**	Radical scaveging, active against isocitrate lyase
*S. thunbergii* [82]	**160**,**161**	-

## Abbreviations

1A9	human ovarian cancer
A549	human lung carcinoma
Aβ25–35	amyloid-peptide fragment 25–35
CDC25B	cell division cycle 25 homolog B
DPPA	1,1-diphenyl-2-picrylhydrazyl
DPPH	1,1-diphenyl-2-picrylhydrazyl
ECD	electronic circular dichroism
EC	Effective concentration
ED	effective dose
EGCG	epigallocatechin gallate
GSH	glutathione
HCT8	human ilececal cancer
HIV-	human immunodeficiency virus
HL60	promyelocytic leukemia cells
HOS	human bone tumor
HSV-1	herpes simplex virus 1
IC	inhibitory concentration
LAR	leukocyte antigen-related phosphatase
LXR	liver X receptor
MCF-7	breast adenocarcinoma
MIC	minimum inhibitory concentration
MRSA	Methicillin-resistant Staphylococcus aureus
P388	mouse lymphocytic leukemia
PC3	human prostate cancer
PPARs	Peroxisome proliferator-activated receptors
PTP1B	protein tyrosine phosphatase 1B
PTPs	protein phosphatases
ROS	reactive oxygen species
SHP-1	src homology phosphatase-1
SHP-2	src homology phosphatase-2
SH-SY5Y	neuroblastoma cell line
SK13	Gram-positive spore-forming bacteria requiring Mn for growth
TCPTP	T-cell PTP
